# TiO_2_ Nanotubes Alginate Hydrogel Scaffold for Rapid Sensing of Sweat Biomarkers: Lactate and Glucose

**DOI:** 10.1021/acsami.1c11446

**Published:** 2021-08-02

**Authors:** Udara Bimendra Gunatilake, Sandra Garcia-Rey, Edilberto Ojeda, Lourdes Basabe-Desmonts, Fernando Benito-Lopez

**Affiliations:** † Microfluidics Cluster UPV/EHU, Analytical Microsystems & Materials for Lab-on-a-Chip (AMMa-LOAC) Group, Analytical Chemistry Department, University of the Basque Country UPV/EHU, Barrio Sarriena s/n, 48940 Leioa, Spain; ‡ Microfluidics Cluster UPV/EHU, BIOMICs Microfluidics Group, Lascaray Research Center, University of the Basque Country UPV/EHU, Avenida Miguel de Unamuno, 3, 01006 Vitoria-Gasteiz, Spain; § Bioaraba Health Research Institute, Microfluidics Cluster UPV/EHU, Avenida Miguel de Unamuno, 3, 01006 Vitoria-Gasteiz, Spain; ∥ BCMaterials, Basque Center for Materials, Applications and Nanostructures, UPV/EHU Science Park, 48949 Leioa, Spain; ⊥ Basque Foundation of Science, IKERBASQUE, María Díaz Haroko Kalea, 3, 48013 Bilbao, Spain

**Keywords:** TiO_2_/alginate, hydrogel, biosensing scaffold, sweat biomarkers, lactate, glucose, sweat sensing, paper

## Abstract

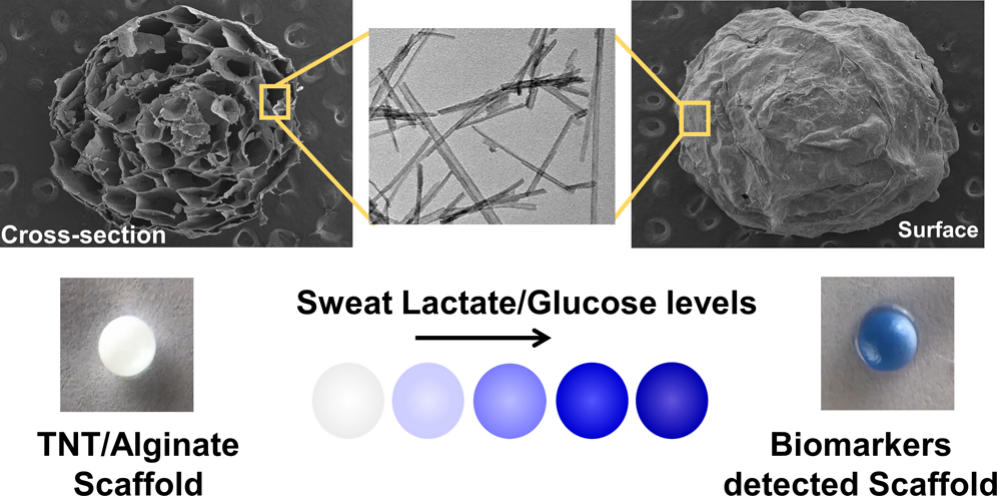

Versatile sensing matrixes are essential for the development of enzyme-immobilized optical biosensors. A novel three-dimensional titanium dioxide nanotubes/alginate hydrogel scaffold is proposed for the detection of sweat biomarkers, lactate, and glucose in artificial sweat. Hydrothermally synthesized titanium dioxide nanotubes were introduced to the alginate polymeric matrix, followed by cross-linking nanocomposite with dicationic calcium ions to fabricate the scaffold platform. Rapid colorimetric detection (blue color optical signal) was carried out for both lactate and glucose biomarkers in artificial sweat at 4 and 6 min, respectively. The superhydrophilicity and the capillarity of the synthesized titanium dioxide nanotubes, when incorporated into the alginate matrix, facilitate the rapid transfer of the artificial sweat components throughout the sensor scaffold, decreasing the detection times. Moreover, the scaffold was integrated on a cellulose paper to demonstrate the adaptability of the material to other matrixes, obtaining fast and homogeneous colorimetric detection of lactate and glucose in the paper substrate when image analysis was performed. The properties of this new composite provide new avenues in the development of paper-based sensor devices. The biocompatibility, the efficient immobilization of biological enzymes/colorimetric assays, and the quick optical signal readout behavior of the titanium dioxide nanotubes/alginate hydrogel scaffolds provide a prospective opportunity for integration into wearable devices.

## 1 Introduction

Real-time sweat analysis opens a noninvasive route to gather valuable information on the variability of biomolecule and ion concentrations over time, where sodium, chloride, potassium, calcium, ammonia, glucose, and lactate are important parameters used to monitor sports performance and health.^1−3^ For instance, lactate is an important biomarker, acting as a vital metabolite in the anaerobic metabolic pathway.^4^ Blood lactate is usually monitored during physical performance since its production is activated by the anaerobic metabolism to provide the required energy to the body by glucose breakdown.^4−6^ Interestingly, it has been demonstrated that sweat lactate increases with blood lactate levels after intense workouts or physical activities.^7,8^ In the same way, glucose is a vital metabolite found in sweat that can be correlated to blood glucose too.^9^ If uncontrolled, it leads to diabetics due to high glucose levels, reaching severe medical complications.

Biosensors have attracted scientists’ attention due to their potential to change classical medical diagnosis and enable novel health-monitoring concepts.^10,11^ Miniaturized biosensors have been recently developed, where electrochemical transductors are widely used due to their low production cost, portability, and ease of operation.^5,12−17^ However, a power-free, simple signal reading phenomenon is essential for real-time sweat analysis wearable devices. Optical detection, and in particular colorimetric sensing, provides simpler signal readout capabilities. It offers rapid quantification of certain analytes, measuring their color variation by absorbance measurements or analyzing color parameters, such as RGB or HUE, by image analysis.^15,18−20^ Thereby, colorimetric detection of biomarkers in sweat becomes simple, cheap, and easily implementable in wearable devices.^18,21^

In this regard, researchers have investigated different sensing platforms for colorimetric sweat biomarker detection. Paper is one of the most commonly used matrixes for colorimetric analysis in wearable microfluidics due to its low cost, biodegradability, biocompatibility, flexibility, and reduced weight. For instance, Mu et al. reported a paper-based skin patch pH test paper device, based on an anion exchanger.^11^ Rogers’ research group reported a wearable microfluidic device for the capture, storage, and analysis of sweat components by integrating a paper matrix to immobilize the colorimetric assays.^18^ Later, Wang et al. developed a chitosan-modified paper platform to detect uric acid and glucose, which showed the use of biocompatible matrixes in this type of device.^22^ Recently, the development of textile-based sensors is on-demand due to their compatibility with human skin and to the increase of the smart-textile market. The work from Promphet et al. is worth mentioning, which fabricated a chitosan-modified cotton textile platform to detect pH and lactate,^19^ and the device presented by Xuecheng et al., a skin-mounted band with superhydrophobic–superhydrophilic microarrays, to be used as a sensor platform for sweat sensing.^23^ However, long-time efficient and effective immobilization of enzymatic biological and/or colorimetric assays, with high loading capacity and rapid sensing capabilities, are still challenging in sweat sensors.

Wearable devices are directly in contact with the human skin, and therefore, the biocompatibility of the sensor is essential when designing sensor scaffolds.^24^ In this regard, alginate is a biocompatible polysaccharide material, which cross-links with di-cationic metal ions to form hydrogels widely used in the food and the pharmaceutical industries.^25−27^ Because of the hydrophilicity and porosity of the alginate hydrogel, external aqueous fluids are easily absorbed by the alginate hydrogel scaffold. Moreover, the water-enriched alginate hydrogel polymer matrix facilitates storing relevant colorimetric assays and catalytic enzymes inside without damaging them.^28^ These properties make alginate, an excellent candidate for the fabrication of biosensors.

On the other hand, titanium dioxide (TiO_2_) is a thermally stable, highly insoluble, nonhazardous, biocompatible, hydrophilic, and photocatalytic inorganic substance, which is used in manufacturing nanostructures.^29−32^ In particular, these titanium dioxide nanostructures have been recently used for biosensing applications.^33−35^ TiO_2_ with 1-D structures, such as nanotubes and nanowires, or fibrous are candidates to enhance the superhydrophilicity and capillary activity of sensor platforms.^30,36^ Therefore, the combination of both alginate hydrogels and the TiO_2_ nanostructures could lead to an upstanding nanocomposite material to be employed as a biosensing platform.

Herein, we present a three-dimensional TiO_2_ nanotube/alginate hydrogel scaffold as a colorimetric biosensor for the detection of lactate and glucose in artificial sweat. The fabrication of the matrix requires two steps: the synthesis of titanium dioxide nanotubes (TNTs) and the cationic cross-linking of alginate to generate a nanocomposite hydrogel. The analytical performance of the sensing scaffold was carried out using spherical-shaped sensors. They were used for the detection of glucose and lactate concentrations in artificial sweat by image analysis. Finally, we integrated the scaffold to a paper substrate by *in situ* hydrogel polymerization to enhance sensing and signal readout performance of the paper matrix.

## 2 Experimental Section

### 2.1 Synthesis and Characterization of TiO_2_ Nanotubes

First, TiO_2_ nanoparticles were synthesized by a precipitation method similar to the one described in ref (37). Briefly, a titanium precursor solution was prepared by adding 5 mL of titanium isopropoxide (97%, Sigma-Aldrich, Spain) to 15 mL of isopropanol (EssentQ, Sharlab, Spain). The solution was transferred to 250 mL distilled water (pH ∼ 2 was adjusted with 1 M nitric acid; 65%, Sigma-Aldrich, Spain) solution under vigorous stirring. Hydrolysis of titanium isopropoxide occurred rapidly, showing a turbid solution. Then, the solution was heated at 60 °C under stirring for 12 h. Afterward, the precipitate was washed with water and ethanol three times and the particles were separated by rotary evaporation at 60 °C under a vacuum.

TiO_2_ nanotubes were synthesized by a hydrothermal method using the synthesized TiO_2_ nanoparticles.^38,39^ About 1.0 g of TiO_2_ nanoparticles was stirred in 20 mL of 10 M NaOH (98%, Sigma-Aldrich, Spain) for 2 h. Then, the basic titania dispersion was transferred to a Teflon-lined hydrothermal autoclave vessel and was heated at 150 °C for 48 h inside the furnace. The precipitate was removed after the vessel was cooled to room temperature and was washed with water and 0.1 M HCl (37%, Sigma-Aldrich, Spain) until the pH of the synthesized TiO_2_ nanotubes reached pH 7–8. Finally, the TiO_2_ nanotubes were separated by rotary evaporation at 60 °C under vacuum.

Transmission electron microscopy (TEM) images of the TiO_2_ nanotubes (in water suspension) were collected from JEOL JEM 1400 Plus (JEOL, Japan) at an accelerating voltage of 120 kV. Scanning electron microscopy (SEM) images of the freeze-dried TNT/alginate scaffolds were recorded by Hitachi S-4800 (Hitachi, Japan) at an accelerating voltage of 10 kV. UV–visible spectra were recorded by Infinite M200 (TECAN Trading AG, Switzerland) microplate reader.

### 2.2 Preparation of the Artificial Sweat Stock Solution

A stock solution of artificial sweat was made using 300 mM NaCl (99%, Sigma-Aldrich, Spain), 40 mM urea (Fisher BioReagent, Spain), 100 mM sodium l-lactate (>99%, Sigma-Aldrich, Spain), and 100 mM d-(+)-glucose (>99.5%, Sigma-Aldrich, Spain) in 100 mL of distilled water. The pH of the solution was adjusted to ∼5.0 (4.8–5.5) with 0.2 M HCl (37%, Sigma-Aldrich, Spain). The desired lactate and glucose concentrations were prepared by diluting the solution with distilled water by considering real sweat conditions.

To perform the assay, 15 μL of the sample was calculated to be enough to surround the full scaffold, without covering it. This volume allowed us to obtain a homogeneous optical signal. However, this volume can be reduced or increased by just engineering the hydrogel shape and dimension and/or the optical detection system.

### 2.3 Fabrication of Alginate/TNT Scaffolds and Analysis

First, 5 mg of TiO_2_ nanotubes was mixed with 1 mL of 1% (w/v) alginate (Sigma-Aldrich, Spain) (1.00 g alginate/100 mL distilled water) for 15 min under sonication, followed by 48 h magnetic stirring (TNT/alginate polymer suspension); the pH of the solution was found to be ∼7.5. For the lactate scaffold, a 5 μL of 0.4 mg mL^–1^ lactate oxidase (LOX) (AG scientific, Spain) solution, a 5 μL of 0.05 mg mL^–1^ horseradish peroxidase (HRP) (Sigma-Aldrich, Spain) solution, and a 5 μL of 3,3′,5,5′-tetramethylbenzidine (TMB) (Sigma-Aldrich, Spain) in dimethyl sulfoxide (DMSO) (>99.7%, Sigma-Aldrich, Spain) (TMB/DMSO, 24:2.25) (w/v) solution were mixed with 30 μL of the TNT/alginate polymer suspension. The suspension was vortexed for 10 s, and the pH of the solution was found to be ∼8.2. Then, 20 μL of the assay mixed polymer mixture was dripped to a 0.4 M CaCl_2_ (93%, Sigma-Aldrich, Spain, pH 7.5) solution to form the TNT/alginate hydrogel scaffold in a spherical shape. The hydrogel scaffold was kept in the bath for 1 min. Then, the scaffold was washed with distilled water for 30 s. The obtained scaffolds were air-dried for 2 min to evaporate the extra surface water. The same protocol was followed to fabricate glucose-sensing scaffolds. In this case, 5 μL of 0.4 mg mL^–1^ glucose oxidase (GOX) (AG Scientific, Spain) solution was used instead of the LOX solution.

To confirm the internal pH of the scaffolds, 15 scaffolds were crushed, stored, and allowed to settle in 1 mL distilled water for 24 h; then, the pH of the solution was measured, obtaining a value of 6.54. This value suggests that the enzymes, within the scaffold, are stable and active since their active pH range is 5.5–9.0, on average; see Table S1 in the Supporting Information.

Then, the scaffolds were tested for lactate and glucose sensing with the desired artificial sweat solutions. About 15 μL of artificial sweat was pipetted onto the TNT/alginate scaffolds, which was placed on a glass slide, and the images and videos of the scaffold color change were captured by a Sony Cyber-shot DSC-RX100 camera over time under controlled light conditions. The images were extracted from the videos at the desired times and analyzed by Image J software.^40^ The intensities of the images were analyzed by mean gray value (black and white, B&W value) 0–255 scale (black = 0, white = 255). Data, statistical, and image analysis were carried out in Excel, Origin Pro 2018, and Image-J.

### 2.4 Scaffold Integration to Filter Paper

Circles of 0.7 cm diameter were printed on cellulose filter paper, Whatman filter paper #1 (Sigma Aldrich, Spain), by a Xerox ColorQube 8570 wax printer, and the wax barriers were generated with an FLC oven, set at 125 °C for 5 min. Next, a 30 μL TNT/alginate nanocomposite with the enzymatic and colorimetric assay (same ratios as in Section 2.3) was drop-casted onto the sensing region of the paper and allowed to absorb on paper for 10 min. Then, the sensing region was dipped in a 0.4 M CaCl_2_ solution for 3 min to generate the scaffold on the filter paper. Finally, the scaffold area was washed with water to remove the excess CaCl_2_. The modified filter paper circles were tested for lactate and glucose sensing with the desired artificial sweat solutions, as explained in Section 2.3.

The glucose sensing scaffolds (both spherical hydrogel and the paper-modified scaffolds) were stored in sealed and moistened conditions at mild temperatures (5–25 °C) for up to 10 days. The sensing readouts of the scaffolds were recorded using a 1 mM glucose artificial sweat solution to determine the stability of the sensor.

## 3 Results and Discussion

### 3.1 Morphological and Structural Characterization of the Scaffold

**Figure 1:**
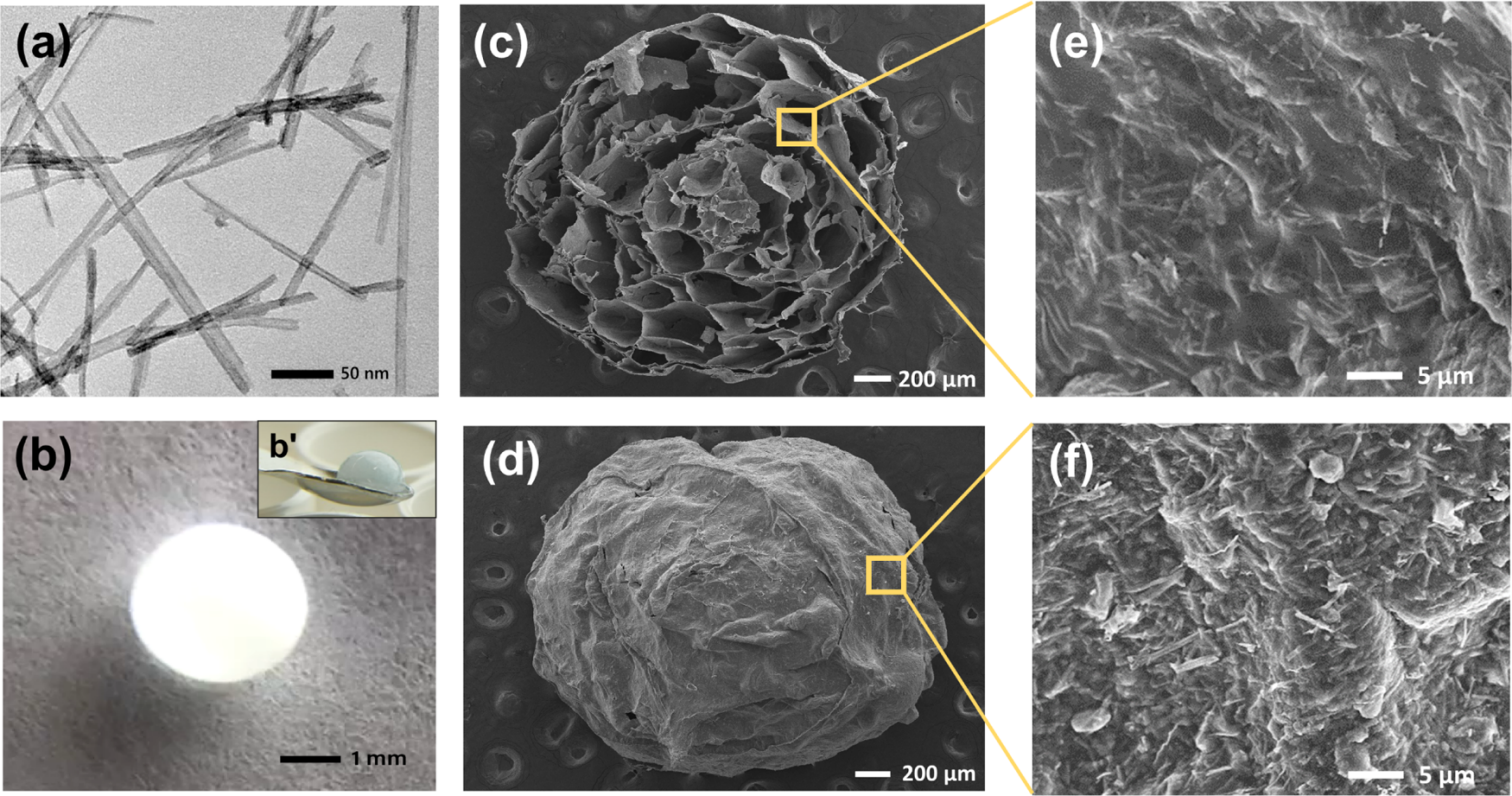
(a) TEM image of the synthesized TiO_2_ nanotubes. (b) Optical image of a TNT/alginate scaffold (spherical bead shape), b′ shows the scaffold in a different angle-sitting on a spatula, slightly blue due to the TMB. SEM images of the freeze-dried TNT/alginate scaffold (c) cross-sectional image and (d) outer surface. (e) High magnification image of the cross section of a petal, and (f) high magnification image of the outer surface.

The TiO_2_ nanoparticles (presynthesized by precipitation) reacted with concentrated NaOH_(aq)_ to form the sheetlike titanate, i.e., layered sodium titanate, which is formed by either dissolution or delamination of titania (reactions 1 and 2). Subsequently, these nanosheets were transformed into nanotube-like structures by exfoliation from the layered sodium titanate (reaction 3). These single or multilayered nanosheets are scrolled and folded to make tubes due to the unbalanced surface energy of the upper and lower sides of the sheets under hydrothermal conditions.^41^ Extreme pressure and temperature conditions inside the hydrothermal vessel led to the formation of different 1D structures like nanofibers, nanorods, and nanowires. Finally, the sodium titanate nanotubes were washed with a dilute HCl solution (0.1 M) to obtain the titanium dioxide nanotubes (reaction 4).
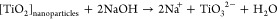
1
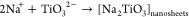
2
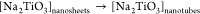
3
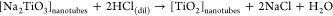
4First, the TNT dimensions and shape were investigated by TEM image analysis; Figure 1a. The images demonstrated that the titanium nanostructures were synthesized in a combination of mainly nanotubes with traces of nanorods and nanofibers (1D structural shapes). Figure S1a,b (Supporting Information) clearly shows a darker intensity in the edges of the nanostructures due to the high electron density of the bending edges of the nanotubes. However, traces of nanorods and nanofibers, Figure S1c,d, were also observed. The average diameter and the length of the TNT were ∼10 and ∼110 nm, respectively. Nevertheless, the synthesis protocol used by us generated a wide range of dimensions including microtubes (see the Supporting Information, Figure S1c,d).

A hanging drop of blended TNT, alginate, and the colorimetric enzymatic assay was generated by a pipette and allowed to drop into a calcium chloride solution bath, generating spherical-shaped hydrogel scaffolds, cross-linking first the surface of the scaffold. The reaction occurred since the dicationic calcium ions and the alginate carboxylate groups cross-linked, following an egg-box model.^42^ Then, the diffusion of calcium ions into the hydrogel allowed the full cross-linking of the scaffold. An optical image of the TNT/alginate scaffold is shown in Figure 1b, exhibiting a 3D spherical shape, which contained the TNT, the bioenzymatic assay, the colorimetric assay, and the CaCl_2_ cross-linked alginate. The diameter of the scaffolds was 3.0 ± 0.2 mm and depended on the volume of the polymer nanocomposite solution used to form the 3D TNT/alginate scaffold. The morphology of the scaffold was studied by SEM. Images of the freeze-dried scaffolds, both the cross-section and the outer surface, are shown in Figure 1c,d, respectively. According to Figure 1c, a microporous honeycomb-like internal cross-sectional structure was seen in the scaffold, where the petallike plates had an average thickness of ∼700 nm. The microporous inter cross-sections were due to vacancies of water-accommodating sites (evacuation of water after freeze-drying) and deswelling of the hydrogel polymer. Interestingly, when comparing the bare alginate scaffolds (without the TNT), Figure S2a,b, with the TNT/alginate scaffolds (Figure S2c,d), higher internal porosity and better-defined internal honeycomb patterns were observed for the TNT/alginate scaffolds. This could be explained as follows: the inorganic phase of TNT, during the cross-linking process, reduced the cross-linking capability of the mixture, thereby increasing the porosity.^43^ TiO_2_ nanotubes contain hydroxyl bonds since they are in moisturized conditions (Ti–OH).^44^ Therefore, TNTs promote hydrogen interactions with oxygen moieties present in the alginate polymer backbone, including the cross-linking carboxylate sites; thus, the cross-linking density between −COOH and Ca^2+^ ions is reduced and the porosity increased. Moreover, the increased hydrophilicity of the mixture due to the TNT^36^ allowed more water to get trapped within the matrix during cross-linking, generating bigger cavities. The enlarged SEM images of the TNT/alginate scaffold surface and the surface of a petal (Figure 1e,f) show submicron-length TNT, demonstrating the immobilization of the TNT, both at the surface and inside the scaffolds. Moreover, the high rugosity observed in both surfaces was an added advantage of the scaffold when used as a biosensor since it increased the hydrophilicity of the materials, and thus, the capacity to absorb liquids inside the scaffold, reducing detection times. Additionally, the internal and external high surface areas of the generated scaffolds helped in immobilizing the enzymes and increasing the number of reactive sites, thereby improving enzymatic catalysis, and thus, both biosensing reactions.

The alginate/CaCl_2_ ratio of 1%:0.4 M was set to be the optimum amount of material needed to keep the hydrogel in a 3D-stable spherical shape. Higher percentages of alginate (over 1%) made the solution too viscous and could not be easily handled during the formation of the scaffold. On the other hand, a lower percentage of alginate and cross-linker resulted in hydrogels with poor spherical shapes due to the low kinetics of the cross-linking while exposing the extruded droplet to the CaCl_2_ bath, mostly because of the reduced amount of carboxylates and Ca^2+^ ions.

The composition of the TNT/alginate in the TNT/alginate polymer suspension was responsible for the mechanical, biosensing, and optical readout properties of the scaffold. The optimization of the composition is discussed in the Supporting Information, Figure S3. The optimum TNT concentration was obtained by varying the amount of TNT (5–25 mg) in the scaffold. The lactate detection assay was performed, and the color intensity and homogeneity of the colorimetric signal were evaluated; see the Supporting Information, SI-3. In view of these results, 0.5% (w/v) of TNT in the alginate matrix (5 mg of TNT in 1 mL of alginate matrix) will be used from now on as the optimized TNT composition for the scaffold: alginate/TNT (2:1). In the hydrogel solution, the concentration of TNT/alginate with respect to CaCl_2_ solution was set to 1% (1.00 g of alginate in 100 mL of water) and 0.4 M, respectively.

### 3.2 Sensing Reaction Mechanism of Artificial Sweat Biomarkers

**Figure 2:**
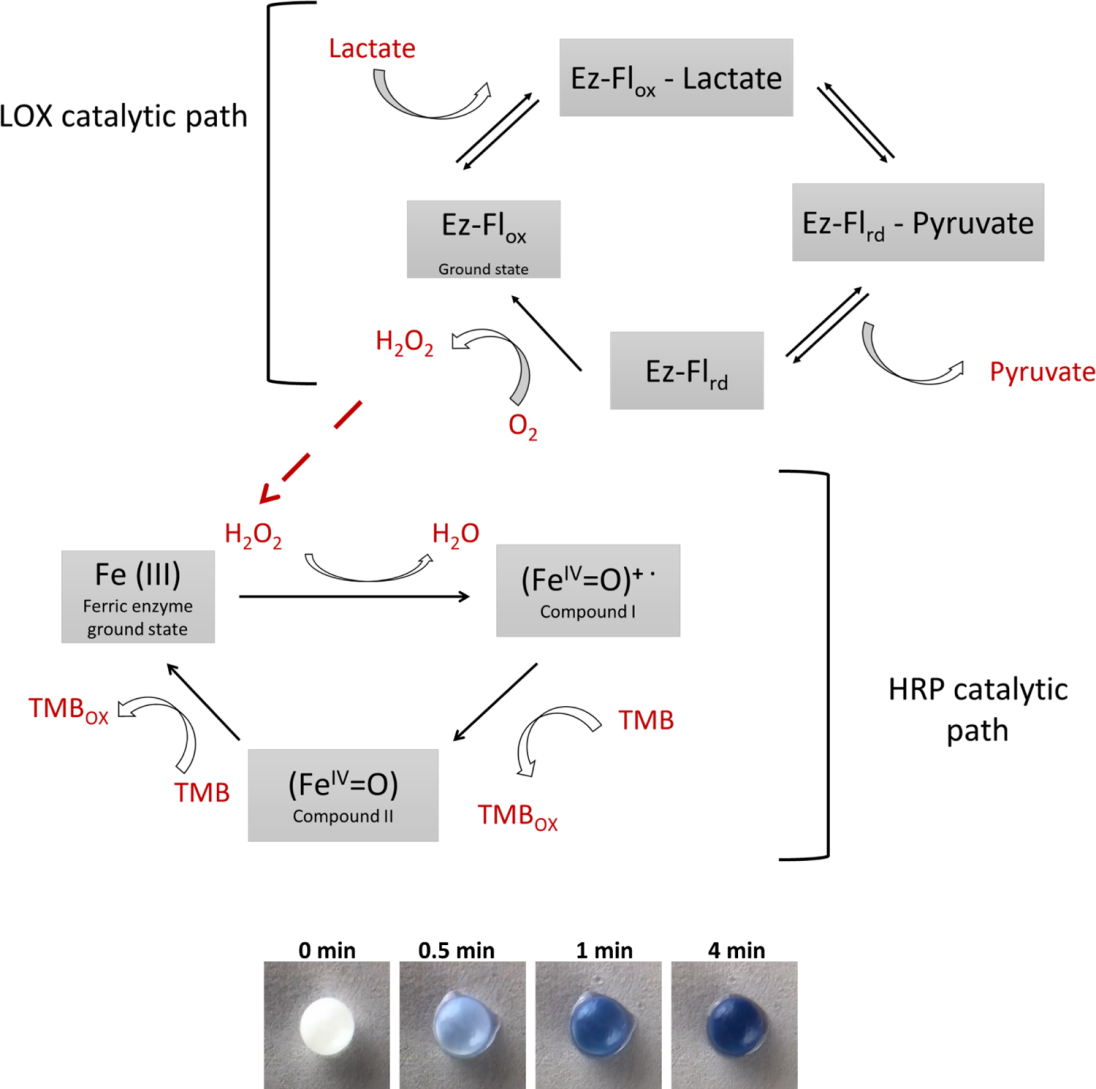
Mechanism of lactate detection under LOX and HRP catalytic pathways (Ez-Fl–flavoenzyme). TNT/alginate scaffolds pictures, at different times, are shown to illustrate the blue color formation (optical signal readout).

LOX and HRP were immobilized in the TNT/alginate scaffold as the catalytic biological enzymes, while the TMB was immobilized as the colorimetric assay (chromophore) for the determination of lactate in artificial sweat samples. The reaction mechanism is shown in Figure 2. In short, lactate is oxidized to pyruvate in the LOX catalysis path, and oxygen (O_2_) is reduced to hydrogen peroxide (H_2_O_2_).^45,46^ Next, the H_2_O_2_ is reduced to H_2_O, and TMB is oxidized to TMB_OX_ changing the TNT/alginate scaffold to blue color in the HRP catalysis path.^47^

In the first pathway, after the lactate entered the TNT/alginate scaffold, a complex is formed between lactate and the LOX enzyme (Ez-Fl_ox_).^46^ The rate of the complex formation was increased by the superhydrophilicity of the TiO_2_ nanotubes, which rapidly incorporated the lactate sample into the scaffold by the capillary effect. In addition, as explained above, due to the high surface area of the TiO_2_ nanotubes, catalytic enzymes had more freedom to spread throughout the scaffold, increasing the reaction sites and getting bonded to the TNTs.^48^ Then, the enzyme–lactate complex (Ez-Fl_ox_–lactate) was converted to the enzyme–pyruvate structure (Ez-Fl_re_–pyruvate) by oxidizing lactate and reducing the LOX enzyme (Ez-Fl_re_). Next, the pyruvate left the reduced state of the enzyme complex. After that, Ez-Fl_re_ was oxidized to its primary state (Ez-Fl_ox_) by releasing electrons that were consumed to reduce molecular O_2_ to H_2_O_2_. In the second cycle, the HRP-mediated redox catalytic reaction was initiated by utilizing H_2_O_2_, which was formed in the first LOX catalytic pathway. In the HRP catalytic path, the H_2_O_2_ is bound to the heme group (Fe^3+^ state) of HRP by forming the heme–H_2_O_2_ hydroperoxo–ferric complex.^49^ Then, with the cleavage of the peroxide bond, heme–H_2_O_2_ proceeded to form H_2_O by reducing H_2_O_2_. Meanwhile, the oxidized form of the heme, compound I, which is an oxoferryl group with a cation radical (Fe^IV^ = O)^+·^ was generated. Then, the oxidized heme returned, via compound II (Fe^IV^ = O), to its native form (Fe^3+^ state) in two steps, accepting electrons from the oxidation of TMB, which turned to blue color (TMB_ox_).^50^ The blue coloration was recorded as the optical signal readout, which was directly proportional to the lactate concentration. The blue product was due to the charge transfer complex of the di-imine-oxidized state and parental diamine of TMB. However, a yellow coloration was expected for the complete oxidation state of di-imine too but, in our case, since the concentration of TMB was very high, the complete oxidation was not promoted. The same type of mechanism can be observed for glucose detection; see Figure S4 in the Supporting Information.

### 3.3 Biosensing Performance of TNT/Alginate Scaffolds

The optical signal readout (blue coloration of the scaffold) was recorded by capturing photos and videos of the TNT/alginate scaffold while subjected to the lactate/glucose biosensing in artificial sweat solutions; see Video S1. The optical readings (color) of the scaffold were analyzed by Image-J software. The color scale was defined by the black and white values (B&W value) of the captured images since there was no color change but an increase in the blue color intensity of the colorimetric signal. The B&W values of the scaffold decreased when the intensity of the blue color increased. Therefore, the signal intensity (blue color) was proportional to the lactate/glucose concentration, but the B&W value of the analyzed images was inversely proportional to the lactate concentration.

**Figure 3:**
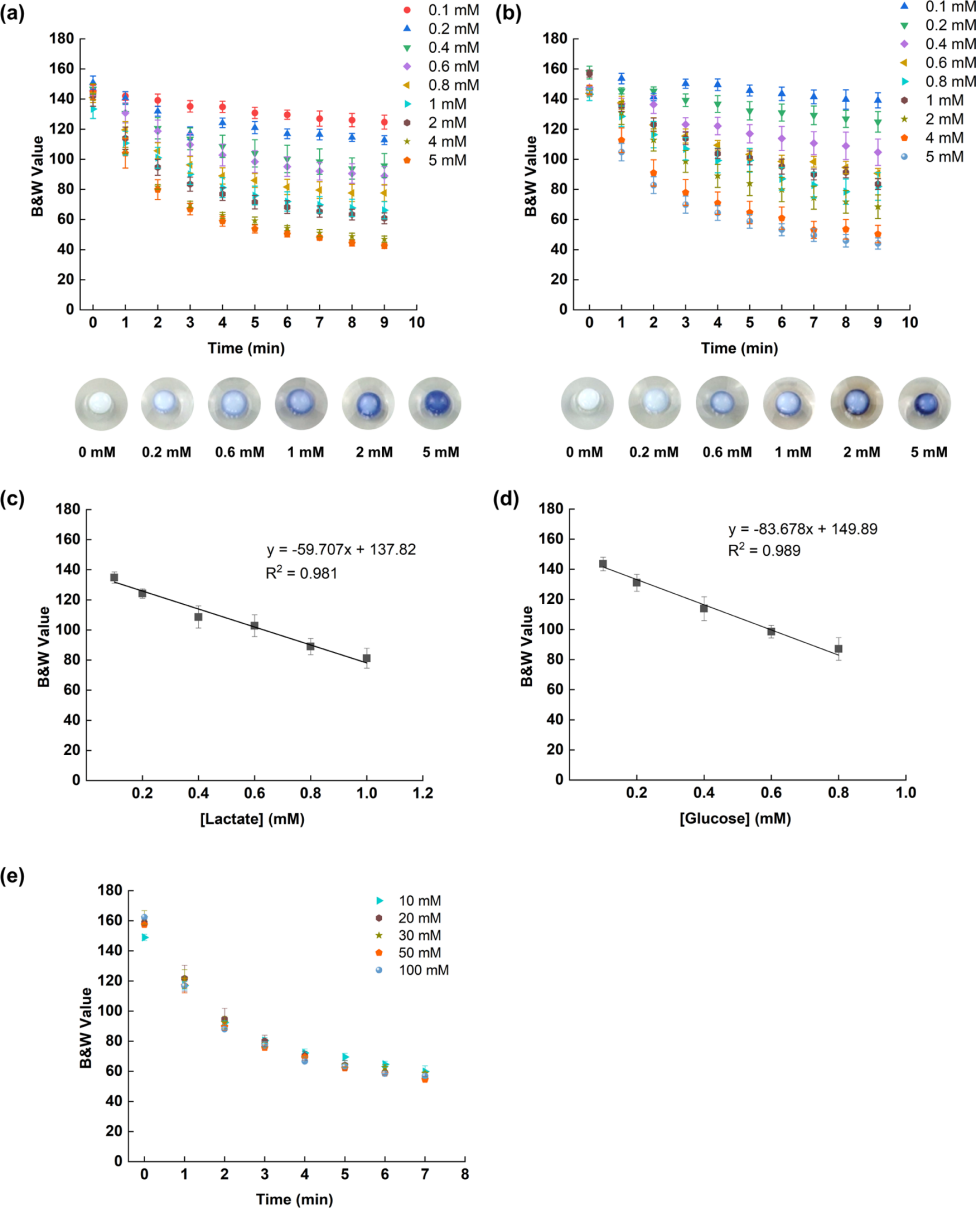
(a) Lactate and (b) glucose detection (blue color development) over time by the TNT/alginate scaffold, lactate, and glucose in artificial sweat (concentration range: 0–5 mM). B&W value was analyzed by Image-J software (max black—0 and max white—255). Calibration plot for (c) lactate and (d) glucose detection in artificial sweat by the TNT/alginate scaffold. Images of the scaffolds at different lactate and glucose concentrations, taken at 4 min time, are shown above the calibration curves. Error bars correspond to mean values ± SD (*n*  =  3). (e) Artificial sweat detection using the scaffold over time (lactate concentration: 10–100 mM).

For the real-time biomarker analysis using wearable devices, sensing time is an important parameter to obtain a useful biosensor. Fast detection times ensure real-time data acquisition and accurate analysis since evaporation, contamination, and degradation of the sample are minimized. Therefore, the sensing time (signal recording time) of the biomarkers using TNT/alginate scaffolds was investigated. The assay was performed with TNT/alginate scaffolds at 0.1–5 mM concentration of the lactate solution of artificial sweat. The color variation was determined following the protocol presented in Section 2.3; Figure 3a for lactate and Figure 3b for glucose.

As shown in Figure 3, the intensity of the blue color can be differentiated at all concentrations studied, after 4 min for lactate and 6 min for glucose. Moreover, the blue color started to saturate at those times, increasing the error of the signal reading (image of the scaffold). Hence, the sensing time was determined to be 4 min for lactate and 6 min for glucose at the colorimetric assay conditions studied. Moreover, the detection time of artificial sweat biomarkers for the bare alginate scaffolds (without TNT) was tested in the same way (see the Supporting Information, Figure S6). The obtained detection time was 12 min, obtaining a much lower response (3× for lactate and 2× for glucose) compared to the TNT/alginate scaffold.

The detection time was fast for the scaffold configuration. Siripongpreda et al.^51^ reported a bacterial cellulose-based hydrogel scaffold with a signal readout at 10 min, while Rusell et al.^52^ reported a poly(ethylene glycol)-based hydrogel with a 10–12 min response time for glucose, which is longer response times compared to our scaffold. TiO_2_ nanotubes promoted the absorption and transport of the sweat biomarkers inside the scaffold reaching the enzymatic centers fast and speeding up the signal observation time, thanks to their superhydrophilicity and capillarity properties. Moreover, the high surface area of the scaffold, both at the surface and inside the matrix, promoted the signal observation times.

The lactate and glucose calibration curves are shown in Figure 3c,d, respectively, and the statistical significance analysis is shown in Figure S5. The signal readouts were recorded for each concentration at 4 min for lactate and 6 min for glucose from the pictures or video taken at those times; see images below the calibration curves. The responses of both biosensors showed good linearity, between 0.1 and 1.0 mM, for lactate and between 0.1 and 0.8 mM for glucose. The dependency between the sensor response and lactate or glucose concentration can be approximated by a linear function with a correlation factor *R*^2^ of 0.981 for lactate and 0.989 for glucose. The statistical limit of detection (LOD) was 0.069 mM with a limit of quantification (LOQ) = 0.23 mM for lactate. In the case of glucose, LOD = 0.044 mM and LOQ = 0.15 mM (LOD = 3*S*/*K*, LOQ = 10*S*/*K*, where *S* is the standard deviation of the blank sample and *K* is the slope of the calibration curve). The linear range and the LOD parameters of the TNT/alginate scaffold for both lactate and glucose are compared in Table S2 to previously published hydrogels. Our sensor scaffold could be directly integrated into real-time glucose sensing devices since it has a quantitative detectable range of glucose, compatible with the glucose ranges of human sweat. However, for lactate concentrations, a dilution factor of the sweat sample would be necessary to decrease lactate concentrations, which normally are high, e.g. 60 mM, which is thus far from our calibration range. It needs to be considered that these values are highly sensitive to the image recording protocol, in which different camera lenses, camera image processing software, light conditions, and object distance or focal distance could alter the obtained results.

The linear behavior of the calibration curve deviated above 1 mM concentrations (see the Supporting Information, Figure S7a, for lactate detection). Therefore, the quantitative detection in both biomarkers was restricted to 1 mM (for lactate) and 0.8 mM (for glucose) when measured at 4 and 6 min, respectively. The signal readout for concentrations from 0 to 100 mM lactate are also shown in Figure S7a, where their values were the same (10–100 mM), within the error (B&W value = 70 ± 5), quantitatively indistinguishable using this image analysis methodology. This behavior can be attributed to the fast color formation at those concentrations. Figure 3e shows the development of the color in the TNT/alginate scaffolds for high lactate concentrations (10–100 mM). The time to reach plateau was found to be very fast for high concentrations of lactate or glucose, less than 1 min, but the values were indistinguishable, regardless of the biomarker concentration. Figure S7b shows the color development for a TNT/alginate scaffold in 10 mM lactate concentration and the pictures of the scaffold at different times during the assay performance. The signal analyzing protocol relies on the intensity analysis of the blue color using the Image-J program. The saturation of the blue color in the scaffold was responsible for the limitation of the quantitative analysis to just low concentrations of biomarkers (0.1–1 mM). However, the quantitative ranges would be shifted by manipulating the colorimetric assay and the enzymatic ratio in the scaffold. On the other hand, as shown in Figure S7b,c, the signal readout recording time could be reduced to detect the color variation at high biomarker concentrations. The scaffolds were also tested with real sweat samples, as described in the Supporting Information, Section SI-12 as a proof of concept. The glucose and the lactate concentrations were determined to be 35 ± 4 and 0.07 ± 0.01 mM using the calibration curves, respectively.

### 3.4 Colorimetric Signal Readout Characterization

**Figure 4:**
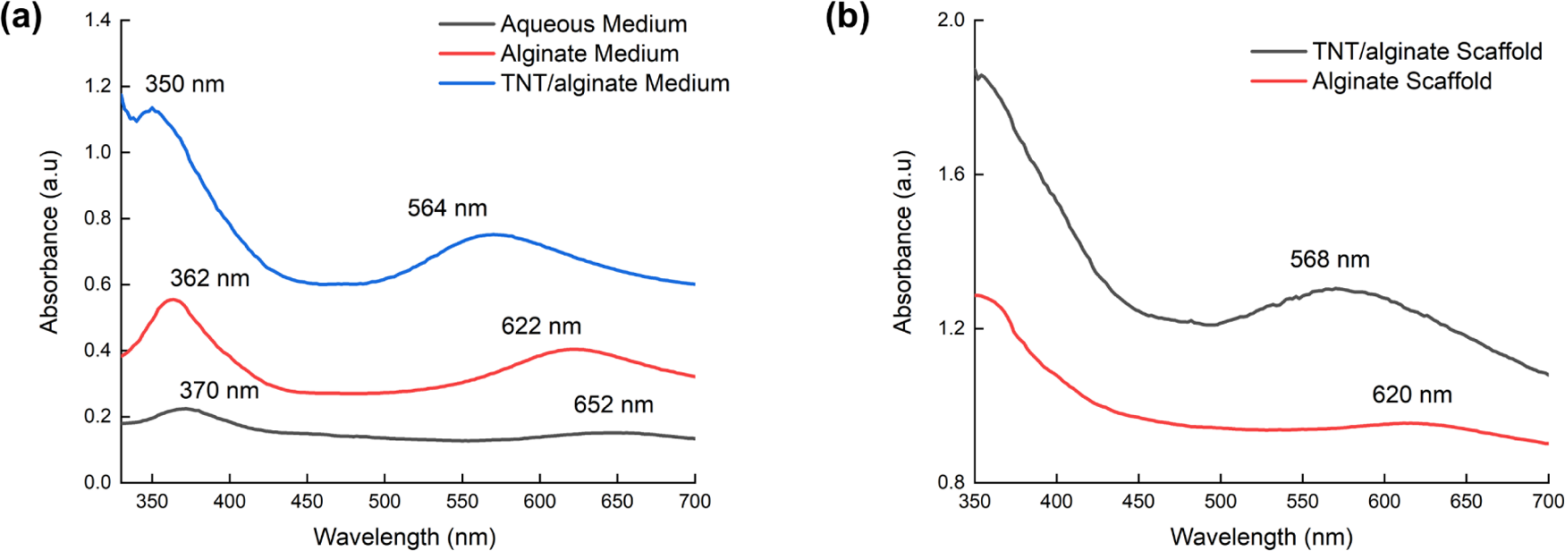
(a) UV–visible spectra of the TMB_ox_ color formation in an aqueous medium, alginate medium, and TNT/alginate medium for 1 mM lactate detection in artificial sweat. (b) UV–visible spectra of the TMB color formation in the alginate scaffold and in the TNT/alginate scaffold for 1 mM lactate concentration in artificial sweat. The UV–visible spectra were recorded from thin layers of hydrogels, placed at the bottom of a transparent 92 well-plate.

The color of the optical signal readout was examined by UV–visible spectroscopy before the hydrogel scaffold was formed, hydrogel solutions, and once the scaffold was fabricated. A 1 mM lactate concentration solution of artificial sweat was added to the samples, and UV–vis spectra were recorded. The color formation of the TMB was analyzed in an aqueous solution (Figure S8a) showing the conventional absorbance peaks of the TMB_ox_, charge transfer complex λ_max_ at 652 and 370 nm. The absorbance of λ_max_ increased with an increase in the lactate concentration from 0.2 to 10 mM in both alginate and TNT/alginate solutions (Figure S8b,c graphs), respectively. However, as shown in Figure 4a, a significant blue shift of λ_max_ was observed from solution (652 nm) to alginate (622 nm), and in the TNT/alginate solutions (564 nm). Similar behavior was observed for the two scaffolds, 620 nm for the alginate scaffold and 568 nm for the TNT/alginate scaffold (Figure 4b). The variation of solvent media/matrix might affect the TMB_ox_ to generate a blue shift in the spectra. Moreover, for the experiments containing TNT, the amine group of the TMB could be physically absorbed to the −OH groups at the TiO_2_ nanotube surface, forming NH–O–Ti hydrogen interactions, which could alter the characteristics of the conjugation system of the TMB_ox_ by contributing to the blue shift of TMB_ox_.

### 3.5 Study of the Possible Interferences Caused by the Catalytic Properties of TNT

TiO_2_ is a photoactive material, which can generate reactive oxygen species under UV irradiation. The generation of electron–hole pairs would, in theory, interfere with the oxidation/reduction mechanism of the assay inside the TNT/alginate scaffolds, acting as a photocatalyst.^53,54^ Therefore, this possible effect was investigated for the TNT/alginate scaffolds using the lactate assay. At normal diffuse light conditions, the TNT/alginate scaffolds retained white coloration for up to 30 min; Figure S9a. This indicated that H_2_O_2_ was not formed without the addition of lactate and that the presence of TNT in the scaffold did not promote self-oxidation of TMB, most probably due to the lack of sufficient energy, in the diffuse light, to generate a photocatalytic effect by the TNT.

Nevertheless, this effect could be triggered during the lactate detection, as the generated blue color of the scaffold could absorb enough light to activate the photocatalytic effect of TNTs. Figure S9b shows the colorimetric signal analysis of the TNT/alginate scaffold in normal diffuse light conditions and in the dark after the addition of 0.4, 1.0, and 5.0 mM lactate. The B&W values of the TNT/alginate scaffold obtained after 4 min did not show any significant difference, within the error, revealing no considerable photocatalytic effect in our experimental conditions.

Recently, TiO_2_-based nanostructures and nanocomposites have been reported to be able to mimic peroxidase activity.^55,56^ Therefore, we investigated this effect by measuring the TMB oxidation activity, without the HRP, in the TNT/alginate scaffolds, under our experimental conditions. Figure S9c presents the images of TNT/alginate scaffolds (lactate assay) for two lactate concentrations, 0.8 and 10 mM, after 0, 4, and 10 min addition of the artificial sweat solutions. The scaffolds without HRP presented no color formation upon the addition of the lactate solutions; thus, the possible catalytic activity of TNT in the scaffolds can be considered negligible under the given experimental conditions.

### 3.6 Scaffold Integration on Paper-Based Sensing Platform

**Figure 5:**
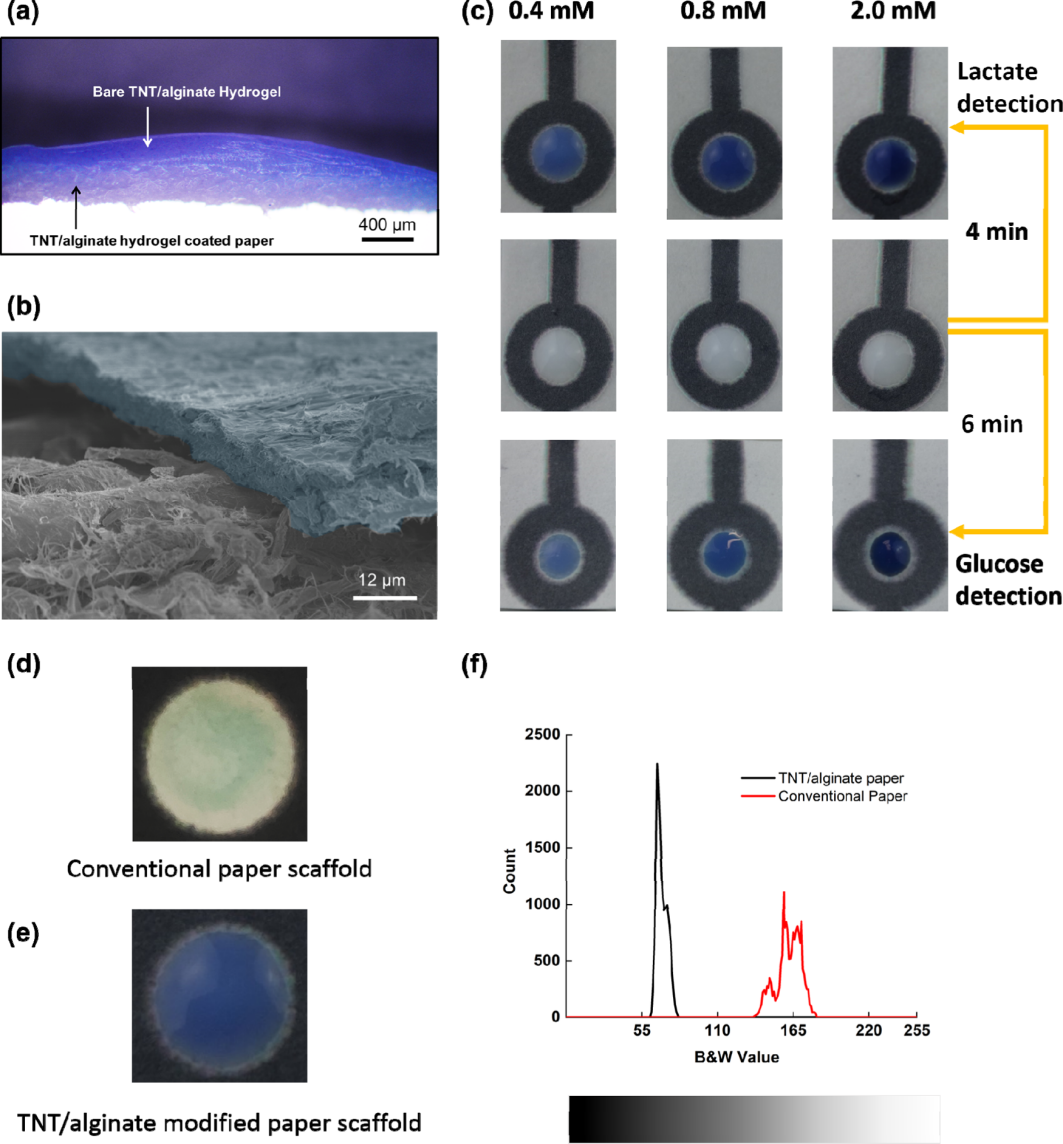
(a) Microscope image of the cross section of the TNT/alginate-modified paper in its hydrated stage. (b) The SEM image of dehydrated hydrogel (blue) on the paper surface (gray). (c) Optical images of the TNT/alginate scaffold-paper surfaces before and after lactate and glucose addition. The white color sensing area represents the modified paper sensing region at 0 min (middle). The scaffold-paper surfaces were checked for 0.4, 0.8, and 2.0 mM lactate and glucose concentrations in artificial sweat. The images were captured at 4 min (lactate) and 6 min (glucose), respectively. (d) Image of a bare paper surface after addition of 0.4 mM lactate in artificial sweat solution captured at 4 min. (e) Image of a TNT/alginate-paper surface after addition of 0.4 mM lactate in artificial sweat solution captured at 4 min. (f) B&W value spreading count from images (b) and (c), analyzed using Image-J software.

Recently, paper-based microfluidics technology is vastly investigated as an easy to implement and low-cost technology to address body fluid analysis at the point of need (wearable devices).^57−59^ Therefore, the possibility to integrate the TNT/alginate hydrogel scaffolds in a paper format as well as the performance of the integrated scaffold was investigated. The TNT/alginate hydrogel was immobilized on the paper as described in Section 2.4. The scaffold was physically bonded to the paper substrate, generating a stable bond between the paper fibers and the hydrogel matrix under moisture and hydrated conditions as shown in Figure 5a. In this case, hydroxyl groups in cellulose can bond with the −OH/–COOH in alginate, mostly by polar–polar and H-bonding interactions. However, that bond was found to get weaker while drying the hydrogel scaffold. The different contraction coefficients of the cellulose and the TNT/alginate scaffold, while dehydrating, weaken the bond as a consequence of the mechanical tension, delaminating the hydrogel; see the SEM image in Figure 5b. The performance of the scaffold-paper substrate was investigated for the lactate and the glucose assays using artificial sweat; Figure 5c. Images were captured at different times, 4 min for lactate and 6 min for glucose, using three concentrations (0.4, 0.8, and 2.0 mM). Moreover, the calibration curves for both lactate and glucose are shown in Figure S10, Supporting Information. The quantitative linear detection range did not significantly deviate from the values obtained for the TNT/alginate scaffolds, in both lactate and glucose. However, the detection range in glucose was extended to 1 mM from 0.8 mM.

A homogeneous optical signal readout was obtained in the scaffold-paper substrate when compared with performing the same assay in the bare paper. In conventional paper substrates, a high percentage of sensing assay loading is limited due to the inherent wettability properties of the paper fibers.^60^ By adding the TNT/alginate scaffold to the paper substrate, the swelling capability of the scaffold, liquid intake, and the porous character of the alginate, permitted a fast absorption of the liquid sample and a homogeneous distribution toward the whole surface of the sensor. Moreover, the high surface area of the TNT, as explained before, generated extra reaction sites for the assay inside the scaffold-paper substrate. An image of a conventional paper substrate, which was used to detect 0.4 mM of lactate is shown in Figure 5d and compared with the same experiment but using the scaffold-paper substrate; Figure 5e. In many microfluidic paper-based analytical devices, coffee ring effects and low homogeneity of the color generated on the sensing areas are common drawbacks that decrease the sensitivity and the precision of the analytical measurement.^61−63^ However, in the scaffold-paper substrate, these effects were minimized. A comparison between the color spreading of the B&W values over the whole paper surface obtained from Figure 5d,e showed a narrower spread of color in the scaffold paper (21 B&W values, 61–82 range) than in the bare paper (45 B&W values, 137–182 range), approximately 2 times higher for the paper, demonstrating the lower homogeneity of the sensing area of the bare paper; Figure 5f. Moreover, the intensity of the color was greatly improved when using the scaffold paper; in Figure 5f, the mean B&W value of the scaffold paper was lower (69) compared to the bare paper (161), while the evaporation time of the sensing area was substantially increased, allowing the acquisition of the results for longer periods of time without increasing the error of the measurement.

### 3.7 Stability of the TNT/Alginate Scaffold

The dehydration of the hydrogel reduced the sensing performance since the enzymes lost their activity. To avoid this, the scaffolds were stored in moistened, sealed, and low temperature (5–25 °C) conditions to keep the enzymes and the assays in an active domain. The sensing performance was stable without presenting any significant change even after 10 days of storage; see Section SI-11.

Based on these preliminary observations, it is stated that the TNT/alginate scaffold is a promising composite material, integrable into microfluidic paper-based analytical devices, able to improve the optical sensing performance of the device. Moreover, it shows a bright future to be used in wearable microfluidic devices for sweat monitoring due to its ease of integration and biocompatibility.^18,64^

## 4 Conclusions

In conclusion, we have introduced a novel platform based on a TNT/alginate hydrogel scaffold for lactate and glucose monitoring in artificial sweat. The scaffold was fabricated by immobilizing enzymatic catalytic assays of LOX/GOX and HRP with TMB chromophore in a TNT/alginate nanocomposite. A rapid colorimetric detection (blue color optical signal readout) was observed for artificial sweat biomarkers in the TNT/alginate hydrogel platform. The signal readout recording times (sensing time) of 4 min for lactate and 6 min for glucose were obtained, whereas the detection time for the alginate scaffold (without TNT) was 12 min. The superhydrophilicity and the capillarity of the TNT were found to increase the detection rate of the biomarkers within the scaffold for both biomarkers. Linear calibration curves for the quantitative detection of lactate (concentration range: 0.1–1 mM) and glucose (concentration range: 0.1–0.8 mM) were obtained with acceptable correlation factors. Furthermore, the TNT/alginate scaffold was successfully integrated into a paper substrate to demonstrate the versatility of the TNT/alginate scaffold to enhance the sensing properties of the paper. High biological assay loadings and quick signal responses were obtained, opening new avenues to improve microfluidic paper-based analytical devices by the incorporation of alginate-based materials. Moreover, this biocompatible colorimetric biosensor scaffold is a promising platform to implement the real-time detection of sweat biomarkers in wearable devices.

## Supporting Information Available

TiO_2_ nanotubes synthesis and characterization; SEM images of the scaffolds; optimization of the TNT composition in the scaffold; glucose detection mechanism; statistical significance analysis for lactate and glucose calibration curves; colorimetric glucose and lactate detection on the alginate scaffold; characterization of the colorimetric detection of lactate (0–100 mM); UV–vis characterization of the lactate assay and possible interferences of the TNT on the colorimetric assay; lactate and glucose detection on the TNT/alginate-paper platform; stability of the TNT/alginate scaffold after 10 days; testing the TNT/alginate scaffold for real sweat samples; active pH ranges of the enzymes used in this study, analytical performance comparison of hydrogel scaffolds as lactate and glucose biosensors (PDF)
Video of the performance of a TNT/alginate scaffold after addition of 1 mM lactate solution in artificial sweat (16× speed) (MP4)
