# Supplementary material for: TiO2 Nanotubes Alginate Hydrogel Scaffold for Rapid Sensing of Sweat Biomarkers: Lactate and Glucose
Source: ACS Appl Mater Interfaces. 2021 Aug 2;13(31):37734–45. doi: 10.1021/acsami.1c11446 (PMC8397235; doi:10.1021/acsami.1c11446)
Supplement: Supplementary file 1 — am1c11446_si_001.pdf [file am1c11446_si_001.pdf]

Supporting Information

TiO_2 Nanotubes Alginate Hydrogel Scaffold for Rapid Sensing of Sweat Biomarkers - Lactate and Glucose

Udara Bimendra Gunatilake[a,b], Sandra Garcia-Rey[a,b], Edilberto Ojeda[a,b], Lourdes Basabe-Desmonts[b,c,d,e*], Fernando Benito-Lopez,[a,c,d,*]

[a] Microfluidics Cluster UPV/EHU, Analytical Microsystems & Materials for Lab-on-a-Chip (AMMa-LOAC) Group, Analytical Chemistry Department, University of the Basque Country UPV/EHU, Spain.

[b] Microfluidics Cluster UPV/EHU, BIOMICs microfluidics Group, Lascaray Research Center, University of the Basque Country UPV/EHU, Vitoria-Gasteiz, Spain

[c] Bioaraba Health Research Institute, Microfluidics Cluster UPV/EHU, Vitoria-Gasteiz, Spain

[d] BCMaterials, Basque Center for Materials, Applications and Nanostructures, UPV/EHU Science Park, Leioa, Spain

[e] Basque Foundation of Science, IKERBASQUE, María Díaz Haroko Kalea, 3, 48013 Bilbao, Spain

*Email- fernando.benito@ehu.eus (Fernando Benito-Lopez)

lourdes.basabe@ehu.eus (Lourdes Basabe-Desmonts)

SI-1: Nanotubes synthesis and characterisation

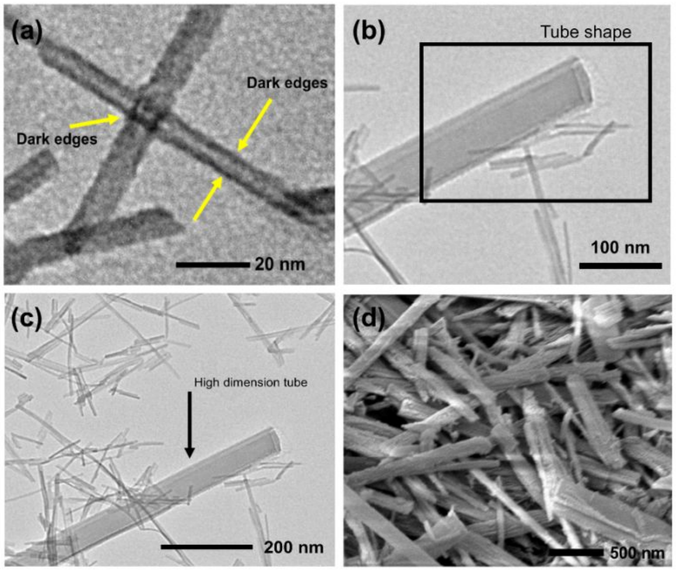

Figure S1: TEM images of the TiO_2 nanotubes (a) dark edges are due to the high electron density in the side edges, thanks to TiO_2 nanosheet bending, (b) cilindrical tube shape, (c) Mixture of TiO_2 nanotubes and a high dimensional (micro) tube, (d) Mixture of TiO_2 nanotubes, SEM image.

SI-2: SEM images of the scaffolds

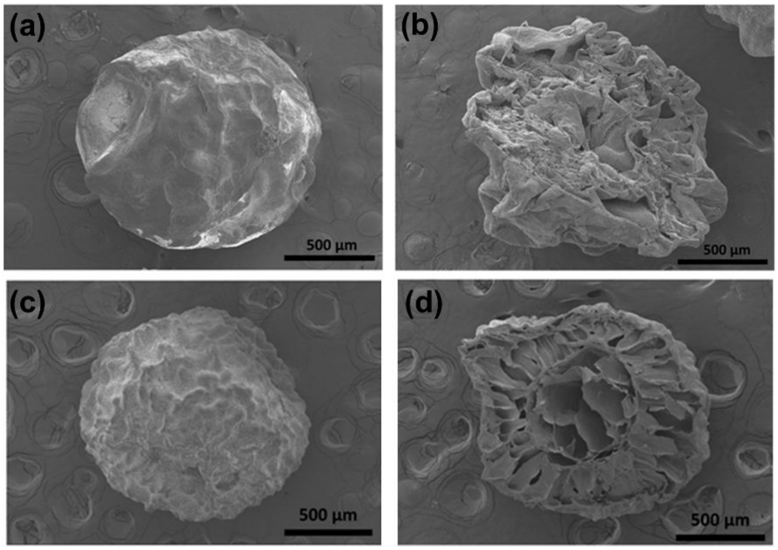

Figure S2: SEM image of freeze dried (a) surface of an alginate scaffold, (b) cross-section of an alginate scaffold (c) surface of a TNT/alginate scaffold, (d) crossection of a TNT/alginate scaffold.

SI-3: Optimization of TNT composition in the scaffold

The optimum TNT composition was obtained by varying the amount of TNT, miligrams, in the alginate matrix and monitoring the colour variation of the scaffolds when a lactate solution was added, see experimental 2.3 section for the fabrication of the scaffolds in the main text, Figure S3a. According to the Figure S3b, the blue colour development is lower over 20 mg TNT than for 5 mg TNT due to the overload of TNT in the scaffold. Bigger error values were obtained at higher TNT concentrations, Figure S3c, therefore, 5 mg of TNT was chosen as the optimal composition for the colorimetric assay. 5 mg of TNT in 1 mL of 1 % alginate presents a 2:1 alginate:TNT ratio thus, one scaffold of 20 µL of alginate contains 0.125 mg of TNT.

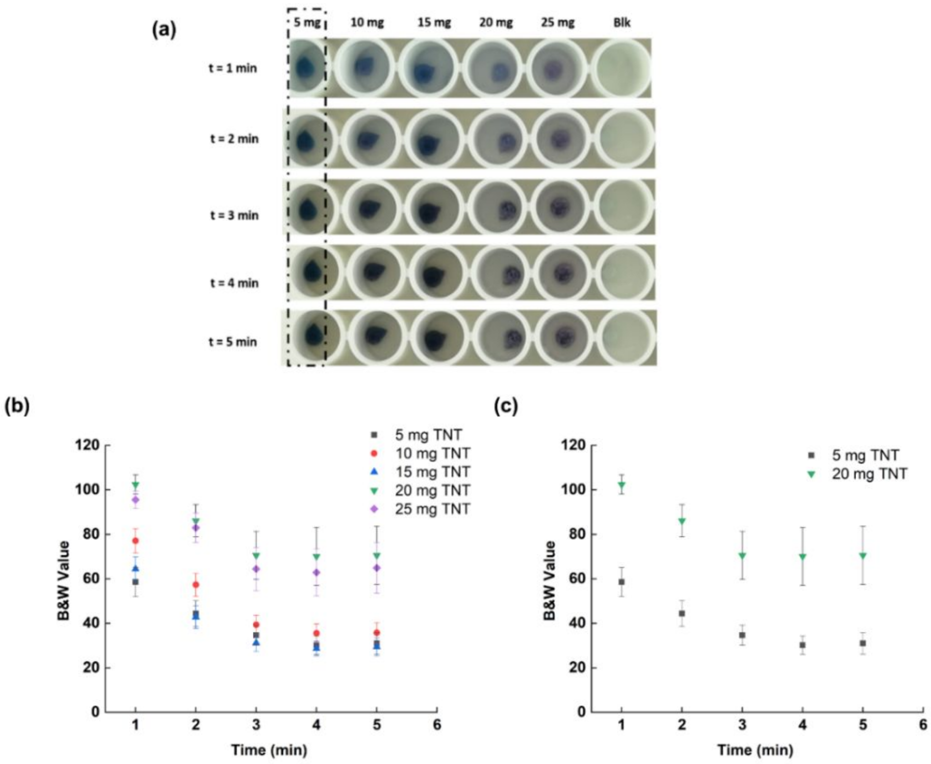

Figure S3 (a) Optical signal redout up to 5 min, for lactate detection (colour development) in TNT/alginate scaffolds at different TNT compositions (5-25 mg of TNT in 1 mL of 1 % alginate solution). (b) 5 -25 mg TNT in 1 mL of 1 % alginate (c) comparison of 5 and 20 mg TNT in 1 mL of 1 % alginate scaffolds. A 25 mM of lactate solution was added to the scaffolds and the B&W values of the scaffold images taken at different times, were analysed by image-J software. Error bars correspond to mean%values ±%SD (B&W value deviation withing the scaffold).

SI-4: Glucose detection mechanism

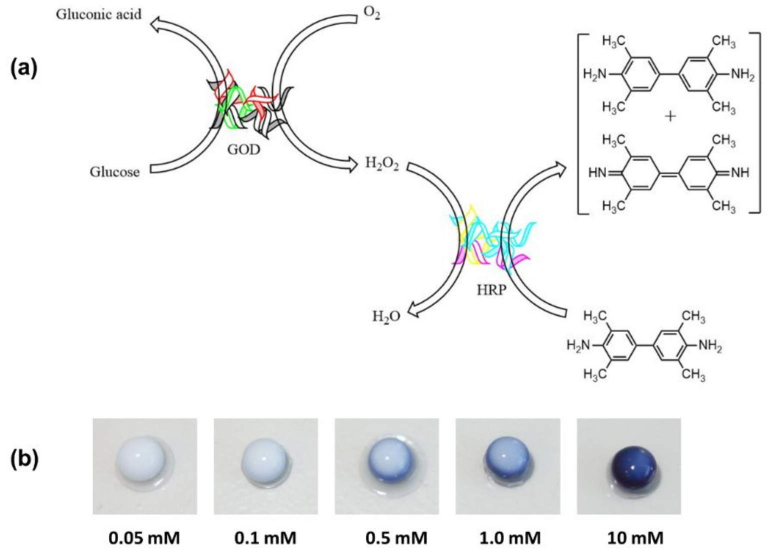

Figure S4: (a) Schematic diagram of the mechanism for glucose detection. First, glucose is oxidised to gluconic acid under GOX (catalyst) and the oxygon is reduced to hydrogen peroxide. Next, the generated hydrogen peroxide is reduced to water while TMB is oxidised, giving a blue colour in the presence of the HRP catalysis. (b) Optical images of the scaffold, 4 min after the addition of 0.05 mM to 10 mM lactate in artificial sweat.

SI-5: Statistical significance analysis for lacatete and glucose calibration curves.

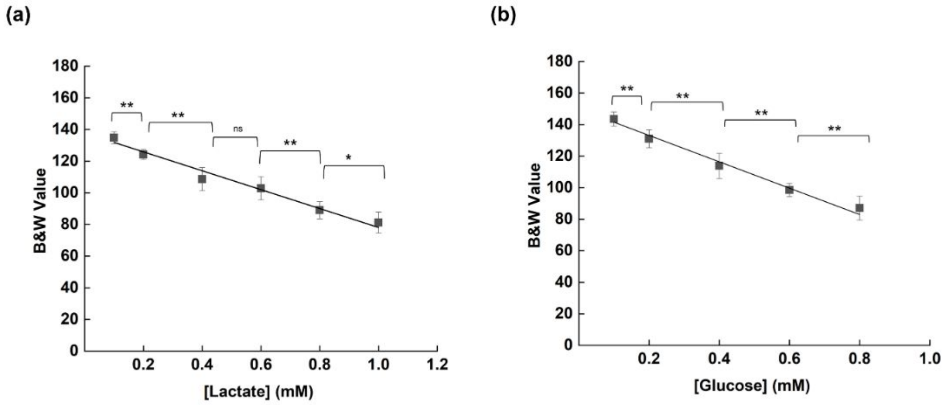

Figure S5 Statistical significance analysis for (a) lactate and (b) glucose calibration curves: one-way analysis of variance (ANOVA), pairwise comparison with Fisher's LSD (with t test). ** $p < 0.05$, * $p < 0.13$, ns- not significant.

SI-6: Colorimetric glucose and lactate detection on alginate scaffold

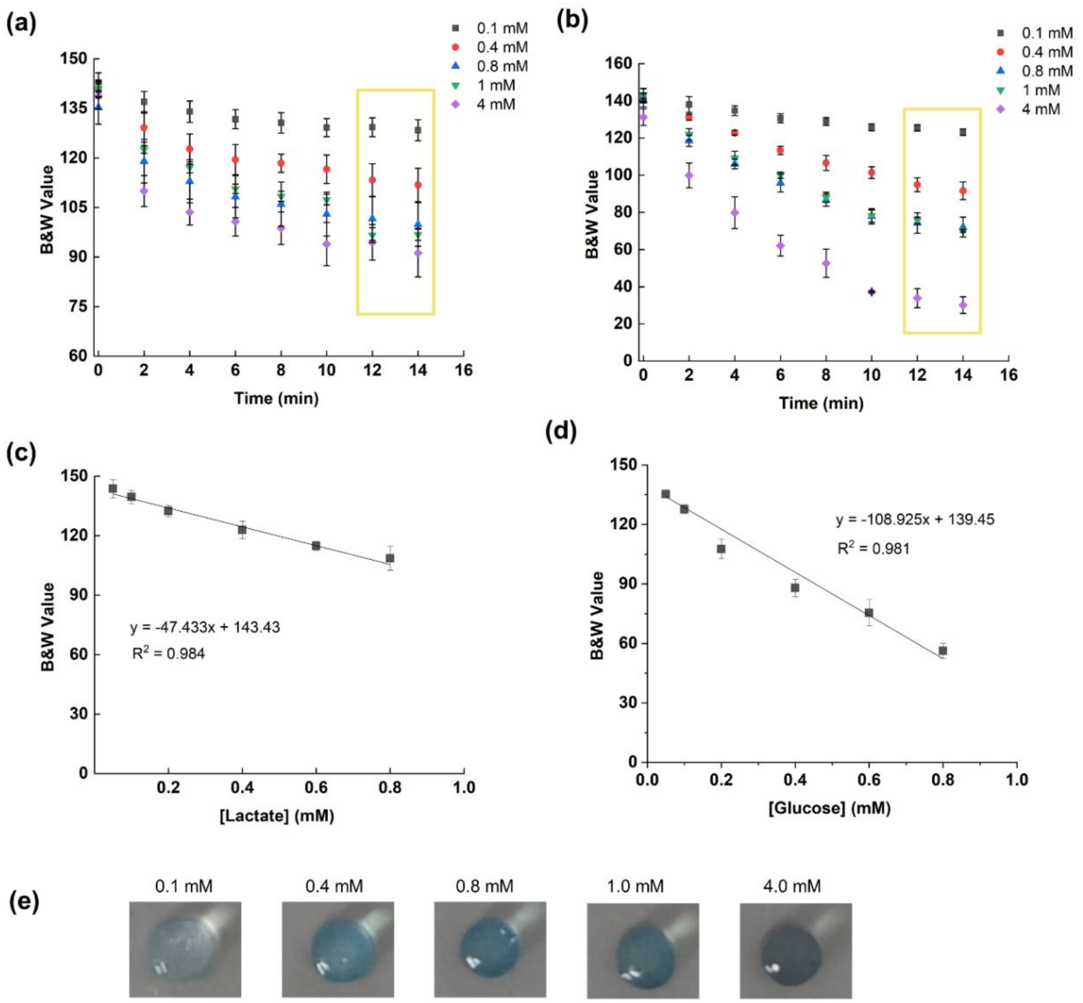

Figure S6: Colorimetric signal formation (colour developmnet) of alginate scaffold for 0.1 to 4 mM of (a) lactate in artificial sweat, (b) glucose in artificial sweat over time, up to 14 min. A

saturation of the signal was observed after 12 min, see the yellow square. (c) Calibration curves for lactate and (d) for glucose in artificial sweat by the alginate scaffolds. A linear regression was obtained in the range 0 to 0.8 mM for both biomolecules. (e) Optical images of the alginate scaffold captured at 12 min in 0.1 to 4 mM lactate artificial sweat solutions. Error bars correspond to mean%values ±%SD (n%=%3).

SI-7: Characterisation of the colorimetric detection of lactate (0-100 mM)

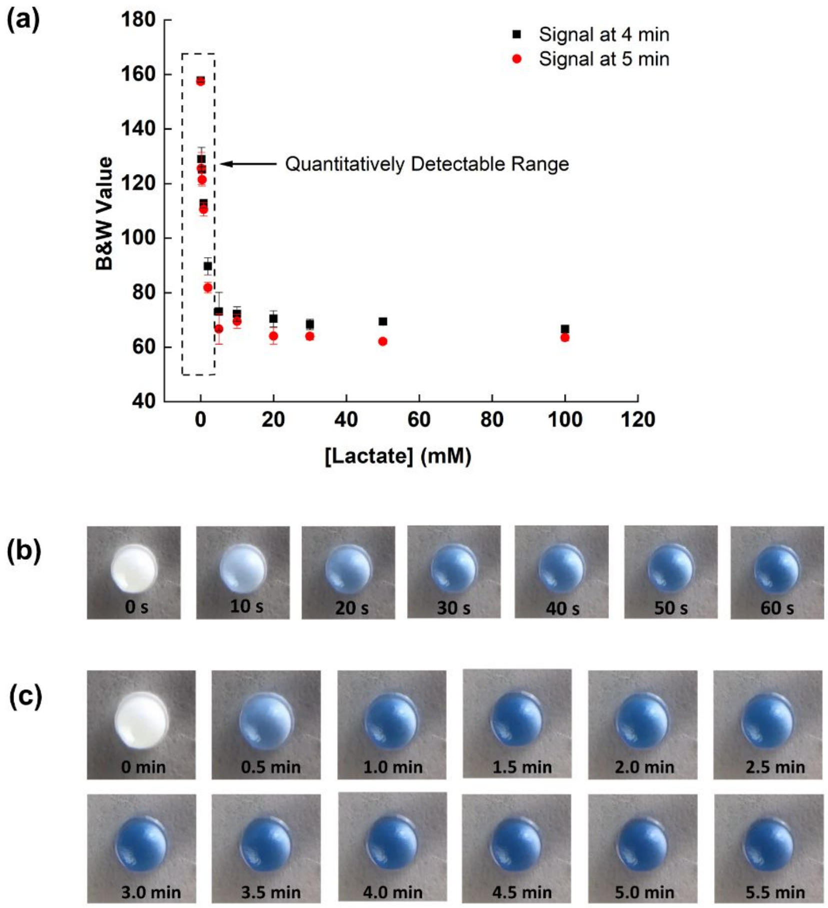

Figure S7: (a) Performance of the TNT/alginate scaffolds for a 0 to 100 mM range of lactate in artificial sweat; the signal was recorded at 4 and 5 min. A linear tendency was observed for 0 to 1 mM lactate concentration range (dashed square). Error bars correspond to mean%values ±%SD (n%=%3). (b) Images of the TNT/alginate scaffolds for the 10 mM lactate detection within 0 - 60 s and (c) 0-5.5 min.

SI-8: UV-Vis characterisation of the lactate assay

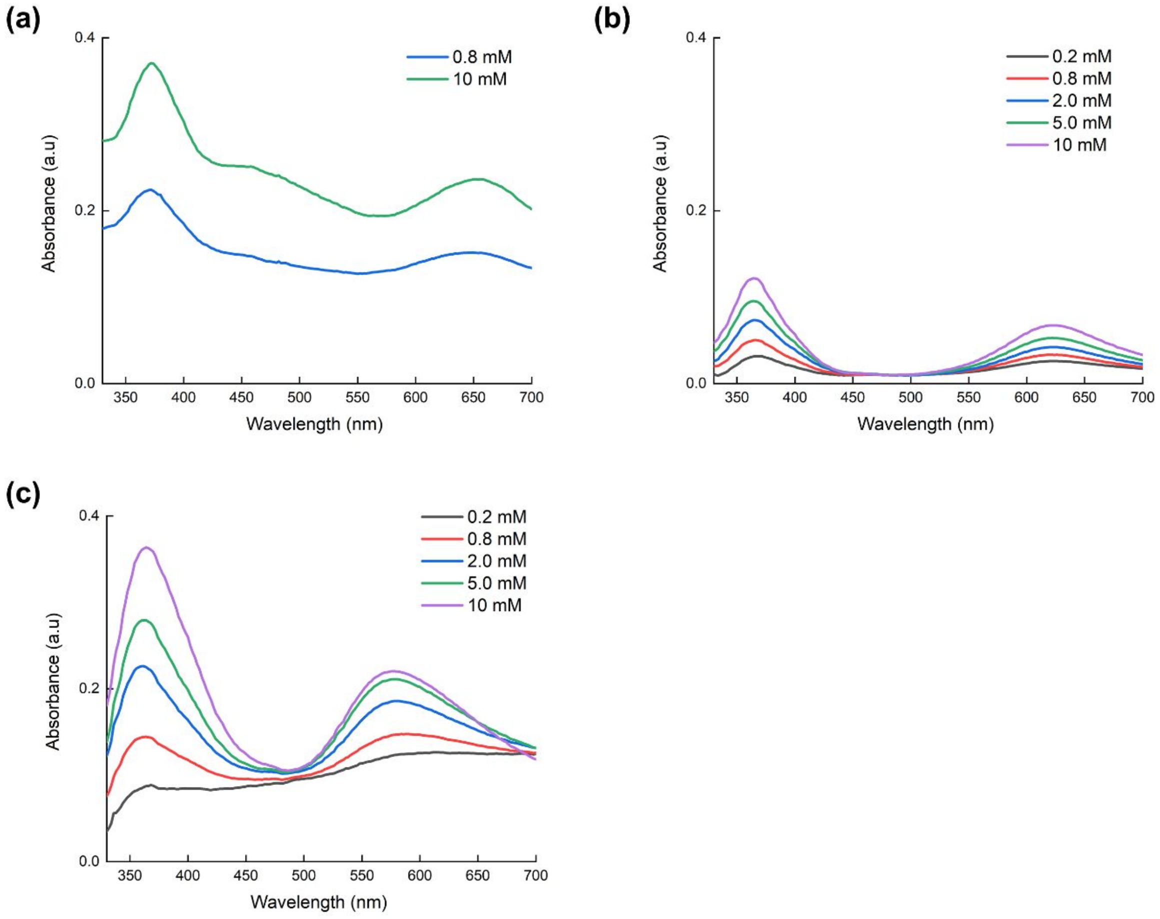

Figure S8: UV-Visible spectra of the TMB colour formation at different lactate concentrations in artificial sweat: (a) in aqueous solution, without the scaffold, (b) in alginate pre-gel solution, (c) in TNT/alginate pre-gel solution.

SI-9: Possible interferences of the TNT on the colorimetric assay

The possible photocatalytic activity of the TNT/alginate scaffolds were investigated by monitoring the assay under controlled diffuse light and dark light conditions. Moreover, the peroxidase activity of the scaffold was investigated with and without the HRP at different lactate concentrations.

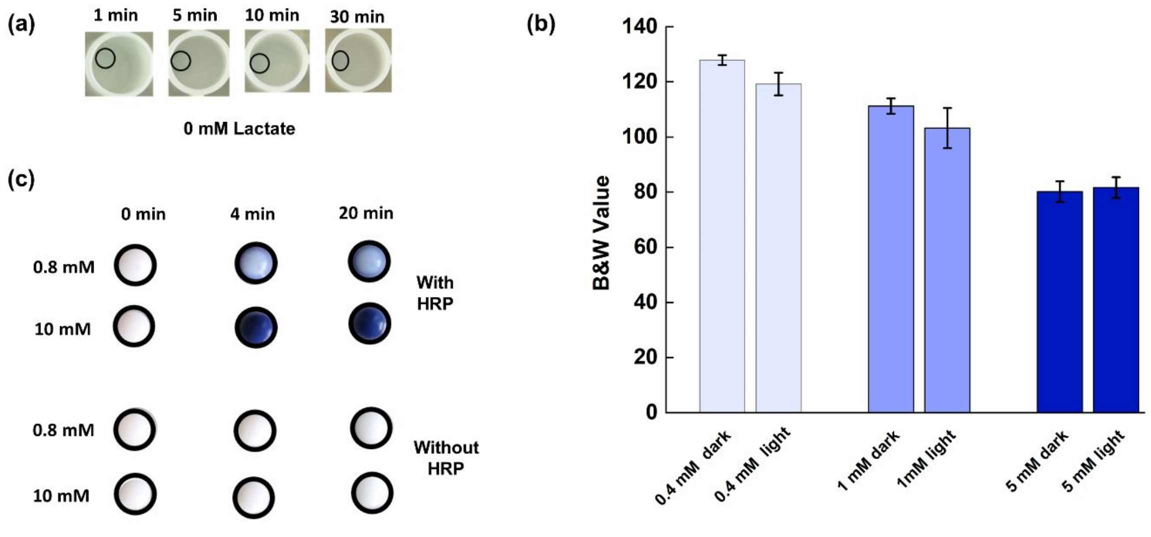

Figure S9: (a) TNT/alginate scaffolds without lactate in artificial sweat, images captured at 1, 5, 10, 30 min. (b) B&W values of the TNT/alginate scaffold activity at 0.4, 1.0 and 5.0 mM lactate concentration under diffuse and dark light conditions. (c) Lactate detection (0.8 and 10 mM) in TNT/alginate scaffolds with and without HRP, images taken at 0, 4 and 20 min. Error bars correspond to mean values ± SD (n = 3).

SI-10: Lactate and glucose detection on TNT/alginate-Paper platform

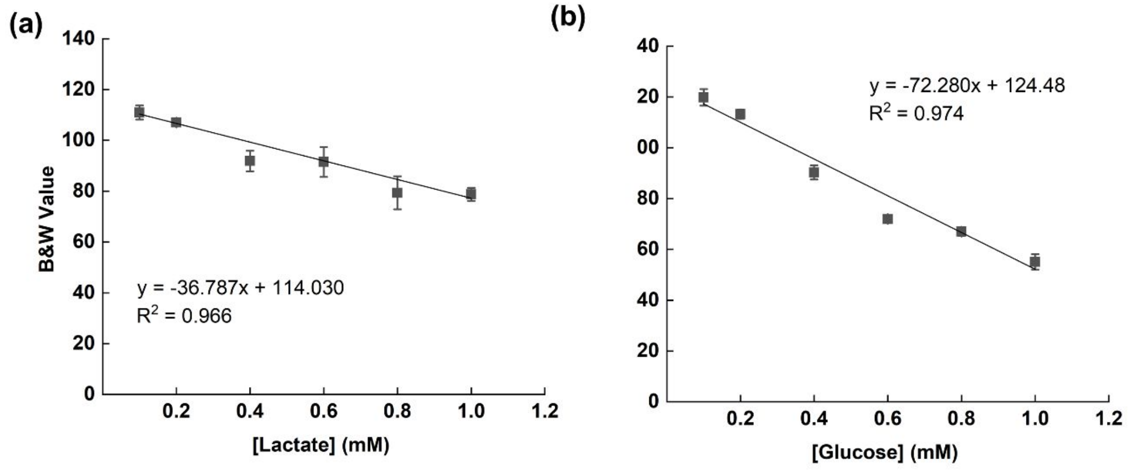

Figure S10: Calibration curves for (a) lactate (signal recoded after 4 min.) and (b) glucose (signal recoded after 6 min) in artificial sweat by the TNT/alginate-Paper platform. A linear regression was obtained in the range 0.1 to 1 mM for both biomolecules. The statistical limit of detection (LOD) was calculated to be 0.195 mM with a limit of quantification (LOQ) = 0.65 mM for lactate. In the case of glucose the obtained values were, LOD = 0.074 mM and LOQ = 0.25 mM; (LOD = 3S/K, LOQ = 10S/K, where S is the standard deviation of the blank sample and the K is the slope of the calibration curve). Error bars correspond to mean%values ±%SD (n%=%3).

SI-11: Stability of TNT/alginate scaffold after 10 days.

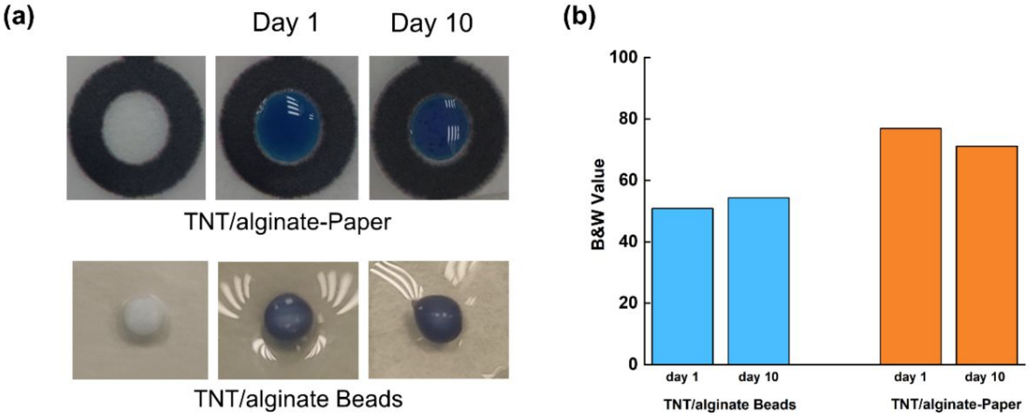

Figure S11: (a) Pictures of the performance of both, TNT/alginate hydrogel in paper and scaffold format, after 1 and 10 days storage at hydrated conditions. 1 mM of glucose was added to the samples. (b) Colour Intensity analysis of the pictures.

SI-12: Testing TNT/alginate scaffold for real sweat samples

Sweat samples were acquired with a pipette from a human (33 years, male, no previous recorded diabetic like complications) while cycling (avg. heart rate 120 bpm, atmospheric temperature 12 ˚C, aerobic phase exercise), from the forehead area of the body; the biomarkers detection was performed at the same time. 15 μL of sweat was directly pipetted to the glucose sensor scaffold while 100 times diluted, with distilled water, sweat was used for the lactate biosensor. This is necessary due to the high concentration of lactate in sweat due to the accumulation of analytes since enough sample volume for the analysis need to be obtained.

The tested scaffolds are shown in Figure S12. The glucose and the lactate concentrations were calculated using the calibration curves (adjusting the optical errors in blanks). 35 ± 4 and 0.07 ± 0.01 mM concentration of lactate and glucose, respectively, were obtained by the TNT/alginate scaffold for the real sweat samples. These values were compared to the values obtained with commercially available gluco (Freestyle freedome lite, USA) and lactate (Lactate Plus, Nova biomedical, USA) meters obtaining a 24.8 mM concertation for lactate. The glucose concentration was not possible to be measure using the commercial available glucometer, since it worked at higher concentration values > 1.2 mM.

It needs to be considered here that, those values are not providing any valuable information about the real analyte concentrations in the sweat sample. Nevertheless, this experiment shows the possibility to use this material as a sensing probe for the detection of lactate and glucose concentrations from real sweat samples, as previously demonstrated using artificial sweat.

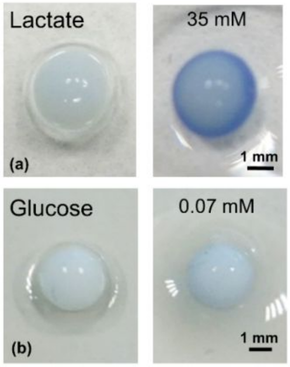

Figure S12: (a) Lactate and (b) glucose tested scaffolds using real sweat, before and after the detection. The response time was set to 4 and 6 min for lactate and glucose, respectively."

SI-13: Active pH ranges of the enzymes used in this manuscript

Table S1: Active pH ranges of the enzymes

Enzyme	Active pH range	Optimum pH
Lactate Oxidase[1]	5.5- 9.5	6.5–7.5
Glucose Oxidase[2]	4.0-7.0	5.5
Horseradish peroxidase[3]	5.0 - 9.0	6.0-6.5

SI-14: Analytical performance comparison of hydrogels scaffolds as lactate and glucose biosensors.

Table S2: Comparison of TNT/alginate hydrogel scaffold to other reported hydrogels as lactate and glucose biosensors.

Biosensing Hydrogel	Biomarker	Linear Range (mM)	LOD (mM)	Ref.
BC/CMC (Bacterial cellulose/ carboxymethyl cellulose)	Glucose	0.00 - 0.50	0.025	[4]
PEG (Polyethylene glycol)	Glucose	0 - 33.3	N/A	[5]
Polyacrylamide	Glucose	0 - 20	0.87	[6]
TNT/Alginate scaffods	Glucose	0.10 - 0.8	0.044	This Work
PAH/PSS deposited Alginate (allylamine hydrochloride)/poly(sodium-4-styrenesulfonate)	Lactate	0 - 1.02	0.0058	[7]
TNT/Alginate scaffolds	Lactate	0.10 - 1.0	0.069	This Work

SI-15: Video of the scaffold performance.

Video S1: Video of the performance of a TNT/alginate scaffold after addition of 1mM lactate solution in artificial sweat (16x speed).

References:

(1) Stoisser, T.; Brunsteiner, M.; Wilson, D. K.; Nidetzky, B. Conformational Flexibility Related to Enzyme Activity: Evidence for a Dynamic Active-Site Gatekeeper Function of Tyr215 in Aerococcus Viridans Lactate Oxidase. *Sci. Rep.* **2016**, *6* (1), 1–13.

(2) Pazur, J. H.; Kleppe, K. The Oxidation of Glucose and Related Compounds by Glucose Oxidase from Aspergillus Niger. *Biochemistry* **1964**, *3* (4), 578–583.

(3) Schomberg, D., Salzmann, M., and Stephan, D. *Enzyme Handbook 7*; Schomburg, D., Salzmann, M., Stephan, D., Eds.; 1993.

(4) Siripongpreda, T.; Somchob, B.; Rodthongkum, N.; Hoven, V. P. Bacterial Cellulose-Based Re-Swellable Hydrogel: Facile Preparation and Its Potential Application as Colorimetric Sensor of Sweat PH and Glucose. *Carbohydr. Polym.* **2021**, *256*, 117506.

(5) Russell, R. J.; Pishko, M. V.; Gefrides, C. C.; McShane, M. J.; Cote, G. L. A Fluorescence-Based Glucose Biosensor Using Concanavalin A and Dextran Encapsulated in a Poly(Ethylene Glycol) Hydrogel. *Anal. Chem.* **1999**, *71* (15), 3126–3132.

(6) Kim, Y.; Namgung, H.; Lee, T. S. Synthesis of a Glucose Oxidase-Conjugated, Polyacrylamide-Based, Fluorescent Hydrogel for a Reusable, Ratiometric Glucose Sensor. *Polym. Chem.* **2016**, *7* (43), 6655–6661.

(7) Biswas, A.; Bornhoeft, L. R.; Banerjee, S.; You, Y. H.; McShane, M. J. Composite Hydrogels Containing Bioactive Microreactors for Optical Enzymatic Lactate Sensing. *ACS Sensors* **2017**, *2* (11), 1584–1588.
